# Sustainability via Active Garden Education: The Sustainability Action Plan Model and Process

**DOI:** 10.3390/ijerph19095511

**Published:** 2022-05-01

**Authors:** Rebecca E. Lee, Jacob Szeszulski, Elizabeth Lorenzo, Anel Arriola, Meg Bruening, Paul A. Estabrooks, Jennie L. Hill, Teresia M. O’Connor, Gabriel Q. Shaibi, Erica G. Soltero, Michael Todd

**Affiliations:** 1 Center for Health Promotion and Disease Prevention, Edson College of Nursing and Health Innovation, Arizona State University, 550 N. 3rd St., Phoenix, AZ 85004, USA; gabriel.shaibi@asu.edu; 2 Institute for Advancing Health through Agriculture (IHA), Texas A&M AgriLife Research, 17360 Coit Rd., Dallas, TX 75252, USA; jacob.szeszulski@ag.tamu.edu; 3 School of Nursing, University of Texas Medical Branch, 301 University Blvd., Galveston, TX 77555, USA; ellorenz@utmb.edu; 4 City of Phoenix Office of Arts and Culture, 200 W. Washington St., 10th Floor, Phoenix, AZ 85003, USA; anel.arriola@gmail.com; 5 College of Health Solutions, Arizona State University, 550 N. 3rd St., Phoenix, AZ 85004, USA; meg.bruening@asu.edu; 6 College of Health, University of Utah, 260 1850 E, Salt Lake City, UT 84112, USA; paul.estabrooks@health.utah.edu; 7 Population Health Sciences, University of Utah, 295 S Chipeta Way, Salt Lake City, UT 84108, USA; jennie.hill@hsc.utah.edu; 8 USDA/ARS Children’s Nutrition Research Center, Department of Pediatrics, Baylor College of Medicine, 1100 Bates St., Houston, TX 77030, USA; teresiao@bcm.edu (T.M.O.); soltero@bcm.edu (E.G.S.); 9 Edson College of Nursing and Health Innovation, Arizona State University, 550 N. 3rd St., Phoenix, AZ 85004, USA; mike.todd@asu.edu

**Keywords:** community, organization, children, intervention study, physical activity, diet, early care and education

## Abstract

Sustainability of intervention programming is challenging to achieve under real world conditions, since few models exist and many studies do not plan far beyond the funding period. Programming content in early care and education centers (ECECs) is often driven by guidelines. However, implementation is very sensitive to contextual factors, such as the setting and implementer (teacher) characteristics. This paper presents the model, definitions, and methodology used for the sustainability action plan capitalizing on a community-based participatory research (CBPR) approach, developed for a multi-site, multi-level garden-based childhood obesity prevention study, Sustainability via Active Garden Education (SAGE). The Ecologic Model of Obesity is applied to develop a sustainability action plan (SAP) and accompanying measures to link early care and education (ECE) environment, the community, policies, and classroom practices to an early childhood obesity prevention program. The SAGE SAP provides an example of how to iteratively evaluate and refine sustainability processes for an obesity prevention intervention utilizing CBPR approaches and will be applied to assess the sustainability of SAGE in a cluster randomized controlled trial. This SAP model can also help inform intervention delivery and scalability within ECECs.

## 1. Introduction

Sustainability of health promotion programming is the concept that policies, programs, and practices can be maintained after an initial intervention period [1,2]. In the case of childhood obesity, effective intervention efforts currently exist [3,4,5]. However, sustaining program effects has been challenging, since the tightly-controlled laboratory conditions of success are not easily maintained or scalable [6,7,8]. Although a critical goal, sustainability is often difficult to attain due to lack of knowledge about the best processes, needed resources, and barriers and facilitators to program institutionalization [9,10,11,12,13]. Prospectively designing and evaluating strategies to improve sustainability are important as they can ensure that the methods used to sustain and disseminate project activities can be replicated [12,14].

In implementation science, approaches that are designed to improve the adoption, implementation, sustainment and/or dissemination of evidence-based interventions are called implementation strategies (hereafter referred to as strategies) [15,16,17]. Strategies can focus solely on one outcome (e.g., sustainment) or multiple outcomes (e.g., implementation and sustainment). They also may be discrete—containing a single component such as a teacher training—or multifaceted, including multiple discrete strategies (i.e., trainings, improving organizational processes, and providing resources) [16]. In short, strategies are all of the support and resources provided to help staff (e.g., teachers, directors) and sites (e.g., Early Care and Education Centers [ECECs]) deliver an intervention [18]. In recent years, researchers have been placing increased emphasis on naming the strategies used to deliver interventions, defining the strategies’ components, and operationalizing how each strategy will work [17], a process that also requires understanding of the broader context in which an intervention is delivered.

Focusing on the sustainability of an intervention is also key to reducing long-term health disparities [12]. Children who experience greater health disparities may live and attend ECECs and schools in contexts where there is even less support and fewer resources to sustain successful health promotion interventions. Sustainability planning is particularly important for improving the delivery of obesity prevention interventions with Hispanic preschool-aged children in the US. Hispanic children participate in less physical activity than their non-Hispanic peers and have higher rates of obesity, diabetes, and other chronic diseases [19,20,21,22,23]. There are also few evidence-based interventions for Hispanic preschool-aged children [24] that occur during the times when they are at ECECs [25]. Generally, school-based obesity prevention interventions have poor long-term effectiveness outcomes due to the numerous barriers and facilitators associated with maintaining the delivery of interventions in this setting [26]. Further, sustainable interventions must be replicable, institutionalized, demonstrate continued benefits, have a supportive community context, and be efficient to deliver [14,27]. Developing sustainability also necessitates significant community and organizational input and buy-in during the formative phases of the intervention [10]. Early buy-in can lead to the transfer of program impact beyond participants to other individuals or environments through linkages (i.e., child at ECEC to parent at home) [28,29], creating a “ripple effect” that is rarely documented or described in the literature [30].

The Ecologic Model of Physical Activity (EMPA) [28,29], which describes the levels and linkages related to physical activity behaviors, has also been applied to dietary habits and nutrition as the Ecologic Model of Obesity (EMO) [31,32]. The EMPA and EMO postulate that health-related behaviors and associated outcomes occur in a dynamic system with external contextual factors that influence individual behavioral choices and decisions directly and indirectly [28,29,31]. These external factors include children’s *micro-level* environments, such as ECECs and homes, their *meso-level* linkages, actors or activities within micro-level environments, such as teachers or family members, and *exo-level* linkages, actors or activities that transfer benefits to other people or places [33]. *Macro*-*level* factors might include societal and cultural norms or policies, as well as, community- and organization-level factors within children’s ecologic milieu. Understanding these contextual elements and improving the ones that influence implementation and sustainability of a childhood obesity intervention at an ECEC can result in carefully and consistently delivered interventions that are executed by motivated and capable individuals. In turn, these interventions can improve an organization’s ability to influence children’s health environments and behaviors. Capitalizing on a community-based participatory research (CBPR) process, the Sustainability Action Plan (SAP) was developed to accompany the Sustainability via Active Garden Education (SAGE) program, a garden-based physical activity and healthy eating intervention for children within ECECs [34,35,36]. The SAGE SAP aims to improve the delivery and long-term maintenance of SAGE. The SAGE SAP evaluation protocols also apply a multilevel framework to investigate ECEC factors that influence intervention sustainability at the teacher level, identify critical processes for improving sustainability at the organization level, and evaluate how a community partnership contributes to program success. The goals of this manuscript are (1) to describe the model and process of the SAGE SAP and (2) describe the evaluation plan for measuring the impact of the SAP on the sustainability of SAGE.

## 2. Method

### 2.1. SAGE Overview and Design

SAGE uses a garden-based curriculum to increase physical activity and improve fruit and vegetable consumption in young children, age 3-to-5 years old, at 24 ECECs to help meet national policy guidelines for ECECs [35]. As previously described, SAGE follows the EMO framework and is implemented in ECECs focusing on *micro-level* environmental changes by installing a garden and working with center directors to guide evidence-based ECEC policy choices. At the *meso level*, teacher training and support is used to deliver an enhanced garden-based curriculum to meet *macro-level* ECEC standards for obesity prevention and early education. Parents are engaged using weekly newsletters linked to garden and classroom activities to enhance *exo-level* transfer of benefits [33].

SAGE includes active learning games and songs that use the plant life cycle as a metaphor for human development. Children and teachers complete garden maintenance and exploration time as a part of the curriculum and to promote garden sustainability. Other curricular activities include three science experiments, mindful eating exercises (“tummy mindfulness”) to sample new fruits and vegetables, and interactive discussion time to reinforce content. All activities are designed to align with National Association for the Education of Young Children (NAEYC) teaching standards.

Garden locations are selected with ECEC directors, maintenance staff, and teachers involved with the delivery of SAGE. If a garden exists already but is not used, efforts are made to breathe new life into the existing build or, if it is not well sited (e.g., inadequate or excessive sun exposure, inadequate drainage), to relocate it. SAGE gardens are sized to maximize access for young children (4’ × 6’) and built using inexpensive, and widely available materials (cinderblocks, soil, trellises). CAB members, teachers, and SAGE staff work together to build the garden. Planting seeds and seedlings is incorporated into the curriculum so that children can participate in this activity. See the garden build protocol in Supplementary File S1.

Following the Childhood Obesity Research Demonstration Project (CORD) sustainability model SAGE’s participatory approach was used to develop the SAP to (1) enhance the potential for SAGE to be *replicable*—repeatable, adaptable or expandable, (2) improve the ongoing *continuation of* SAGE *benefits* from program effectiveness, (3) understand existing policy and organizational structures in order to enhance *institutionalization*, and (4) leverage and expand *community capacity* [2]. SAP processes occur before and during implementation, and the impact of the SAP is evaluated throughout the protocol using a mixed methods approach.

### 2.2. Sustainability Action Plan (SAP) Strategies

**Figure 1:**
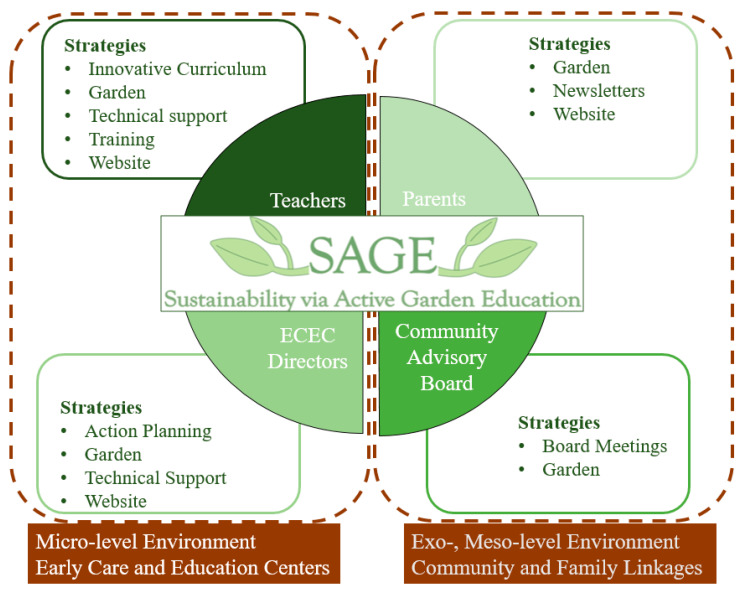
Strategies by stakeholder framed by ecologic level in the SAGE SAP.

The SAP incorporates strategies that encourage community members, ECEC teachers, ECEC directors, and parents to engage with SAGE to help create buy-in, improve program delivery, and develop sustainable processes that improve the ability of the ECEC to maintain SAGE. Strategies were developed to engage each stakeholder group (Figure 1). Processes were created on build and maintain the garden, engage all groups and foster coordination, collaboration, and cooperation between them, to enhance behavioral persistence [37], and in turn, sustainability [2].

*Community Advisory Board Meetings.* The SAGE CAB membership, roles, and responsibilities have been previously described [34,35]. In the context of sustainability, the CAB serves as an important resource and sounding board for SAGE. The SAGE CAB for the SAP comprises local community leaders who represent gardening, early childcare, local and state government, and health communities. The CAB and the scientific team identify and implement short-term and long-term goals for the SAP in bi-monthly meetings. CAB members are paid a modest honorarium of $100 annually, and the chair, $200. The CAB provides important information about community capacity and serves as a bridge between the scientific team and community. Each CAB member serves a one-year term at the end of which, the member is given the opportunity to rotate off or continue for an additional term.

*Adaptive Curriculum Delivery Approach.* The SAGE curriculum will be introduced using a continuous one-hour format two times per week for 12 weeks. However, activities may be completed sequentially or re-ordered, offered all in one go, or throughout the school day to fit different teaching styles and classroom schedules. Moreover, activities may be offered indoors or outdoors to accommodate bad weather (e.g., excessive heat).

*Teacher Training and Technical Support.* Another part of curriculum delivery that promotes sustainability is the use of a “see one, do one, teach one” teacher training method that has been used in health care delivery. The first 12 sessions will be led by the research team over six weeks, with teachers observing and assisting in SAGE delivery. Gradually, teachers will begin to lead portions of sessions during this time. Then, the 12 sessions can be repeated over a second six-week period and led by the teachers with assistance from the research team. Teachers will complete a 90-min training session prior to beginning implementation and a 30-min booster session about five weeks into implementation. Trainings focus on five PRIME principles that reinforce best practices for delivering SAGE: *P*romote positive experiences, *R*oom management, *I*mprovement directed reinforcement, *M*odeling appropriate behaviors, and *E*stablishing garden maintenance. On-demand technical support will be available throughout the study for ECECs and teachers who desire additional help.

*Action Planning Meetings with Directors.* Over the course of the SAGE program, research staff will meet three times for one hour with ECEC directors to develop a plan for institutionalizing SAGE and sustaining it beyond the funding period. ECEC directors will also complete assessments to evaluate resources, barriers, and facilitators for sustaining SAGE. In the first meeting, directors will complete a self-assessment of their ECEC, be given the opportunity to ask questions or voice concerns about SAGE, and prompted to think about long-term sustainability. In the second meeting, after they have more experience with SAGE, directors will review the results from their assessments, discuss how their results compare to other ECECs, and develop an action plan and set goals for improving low-scoring areas. Each action plan will be discussed with the CAB to identify community resources to support each ECEC director’s goals and provide feedback. In the final meeting of the semester, SAGE staff and directors will discuss progress towards achieving their goals, reevaluate and set new goals as needed, and complete assessments a second time.

*Newsletters.* SAGE newsletters are designed to engage parents with the curricula, reinforce curricula content when children are at home, and promote the benefits of the ECEC having a garden to encourage parents to become advocates for SAGE. Twelve newsletters are sent out bi-weekly and are coordinated with the SAGE session that children completed during that week. Newsletters include healthy recipes that parents can prepare at home, physical activities for parents and children to complete together, information about local community events for nutrition and physical activity, fun activities for children to complete (e.g., pictures to color, connect the dots drawings), and pictures of children participating in SAGE at the ECEC. Newsletters are available in English and Spanish [33,36].

*Website.* The SAGE website includes information for directors, teachers, and parents about the curriculum, needed materials, and instructions. Links are provided to videos demonstrating active games, science experiments and the tummy mindfulness exercise (https://sites.google.com/asu.edu/sageasu/home, accessed on 1 April 2022). Periodic blog posts provide additional information about school gardening, ideas for engaging kids and families in physical activities, and simple strategies for eating more fruits and vegetables.

### 2.3. SAP Rollout Process

**Figure 2:**
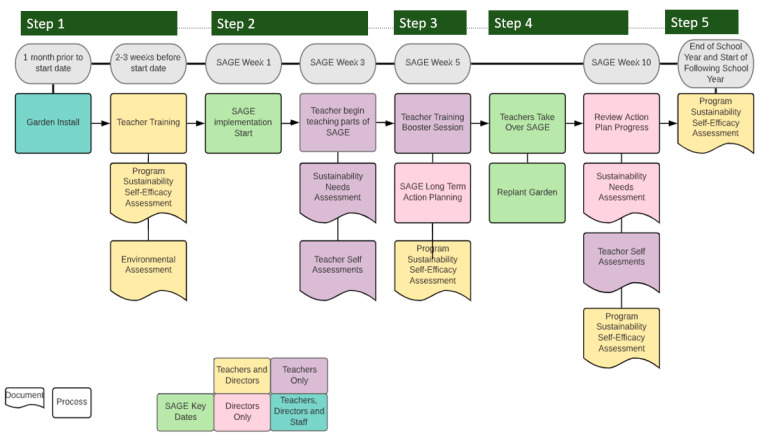
The SAGE SAP steps rollout process and measures by stakeholder.

The SAP commences before the curriculum begins and unfolds in a five-step process, which is also described using the plant life cycle as a metaphor. Figure 2 depicts this process and is color coded by stakeholder. Step 1 focuses on planting the idea of sustainability. Steps 2 and 3 focus on reinforcing and growing interest in the concept of sustainability. Steps 4 and 5 empower ECEC staff to sustain SAGE and transition responsibilities and resources associated with SAGE to the ECEC and community.

*SAP Step 1*. *Planting the seeds: Organizational assessment and training (Pre-Implementation).* Prior to implementation, SAGE staff will meet with teachers and ECEC directors to deliver curriculum supplies and conduct an initial training. This training introduces the PRIME principles (described above), provides a brief overview of the curriculum, and gives the ECEC staff a tour of the garden where they have an opportunity to plant seeds as part of the garden introduction. Fresh fruits and vegetables that represent what might be served as part of the curriculum are sampled during this meeting, and several songs and games demonstrated and played with center staff. During all activities, SAGE staff emphasize how the curriculum can fit within current teaching practices and connect activities to NAEYC standards.

*SAP Step 2. Weed control: Identification of challenges (weeks 1–3).* Once ECEC personnel begin to have some experience implementing SAGE, an assessment will be administered to ECEC directors to investigate process factors associated with implementing the new program. Teachers also complete a self-evaluation. Data from all assessments will be reviewed and used to develop the booster training session in SAP Step 3.

*Step 3. Nurturing the seedlings: Overcoming challenges (weeks 4–5)*. Challenges identified in the assessments will be presented to the CAB, ECEC directors and teachers, and the scientific team to allow all parties to develop a shared vision of how SAGE might be replicated in future classrooms. This step also nurtures community capacity by improving evidence-based knowledge about meeting health behavior guidelines and education standards, improves the potential for continuation of benefits to children as they develop, and identifies current and future resources and changes needed to promote institutionalization of SAGE.

At this step, a booster training session will be conducted with teachers. The booster training is used to provide formalized technical assistance as teachers begin to become the primary implementers—switching from the “see one” phase to the “do one” phase of implementation. The booster training helps teachers consider the best processes to integrate SAGE within their teaching styles and existing curriculum. Reflective listening and brainstorming will be used to generate site- and teacher-specific modifications that might be needed for sustainable delivery of SAGE. Booster sessions will be informal, but generally structured around the sustainability of three pieces of the program: gardens, the tummy mindfulness and experiential eating taste tests, and the curriculum.

*SAP Step 4. Preparing for harvest: SAP implementation (weeks 5–10).* Representatives (director, staff, and teachers) from each ECEC will meet with the research team to review how their center fared on assessments. Weaknesses can be identified with strategies to overcome them, based on realistically considering timelines and priorities. Two or three goals will be set based on areas identified for improvement (e.g., improving center policies, sustaining funding, training staff). SAP implementation progress will be reviewed during CAB meetings, and the CAB suggests helpful community resources. The research team will communicate this information and connect ECEC staff to resources.

At the end of step 4 and the 12-week intervention, assessments will be collected to determine how the SAP affects outcomes. Information collected during this phase will be relayed back to the CAB and ECEC directors. The CAB, ECEC directors, and the scientific team will work together to modify SAGE curricular components and implementation protocols for subsequent cohorts of ECECs that receive the SAGE program.

*Step 5. Harvesting the fruit: Interpreting and disseminating findings*. The SAP rollout process continues for three cohorts, including data collection throughout. Information gleaned can inform changes to the SAP. By using a cross-over design, the organizational assessment, development, implementation, and evaluation of the SAP is repeated 6 times over 3 cohorts. Thus, there is the opportunity to develop a comprehensive and robust SAP if changes are needed.

### 2.4. SAGE SAP Sources of Data

To evaluate the impact of the strategies on each CORD construct and at each level of the EMO, a battery of measures designed to include perspectives and reflect priorities of each stakeholder will be administered. A summary, organized by sustainability construct, is presented in Table 1. At the micro level, teacher exit interviews and ECEC directors surveys are conducted, along with on-site environmental surveys (NAP SACC, PARA), and which are to be triangulated with extensive field notes during implementation. The Wilder Collaboration Factors Inventory is completed annually with our community advisory board. At the meso-level, in addition to relying on teacher interviews, teachers complete a questionnaire, and parents complete a brief survey. Teacher surveys inform the ex0 level. At the macro level, meeting minutes, environmental assessment, and CAB measures will be examined.

To maximize understanding of the efficacy of the SAP, information will be collected from all stakeholders. CAB members will complete a survey evaluating their role with the collaboration. Teachers will complete self-evaluations of their ability to implement SAGE, score parent-teacher interactions that may affect sustainability, rate their self-efficacy for sustaining SAGE beyond the funding period, provide reflections on SAGE implementation after each session that are recorded in field notes, and participate in post-intervention interviews. ECEC will directors evaluate factors associated with program sustainability related to ECEC administration, policies, and procedures, rate how SAGE has impacted their site, score their self-efficacy for sustaining SAGE beyond the funding period, and participate in post-intervention interviews. Parents will complete surveys evaluating the newsletters and rating their interaction [33]. SAGE staff, in coordination with ECEC teachers and directors, will complete assessments to evaluate the physical activity and nutrition environment at the ECEC, as well as record meeting minutes of all meetings with CAB members, ECEC teachers, and ECEC directors. The timing of each assessment is presented in Table 2.

### 2.5. Community Advisory Board Assessments

The *Wilder Collaboration Inventory* is a tool that measures the collaboration between and within groups that represent multiple interests using 40 questions reflecting twenty dimensions: (1) history of collaboration or cooperation in the community; (2) collaborative group seen as a legitimate leader in the community; (3) favorable political and social climate; (4) mutual respect, understanding, and trust; (5) appropriate cross-section of members; (6) members see collaboration as in their self-interest; (7) ability to compromise; (8) members share a stake in both process and outcomes; (9) multiple layers of participation; (10) flexibility; (11) development of clear roles and policy guidelines; (12) adaptability; (13) appropriate pace of development; (14) open and frequent communication; (15) established informal relationships and communication links; (16) concrete, attainable goals and objectives; (17) shared vision; (18) unique purpose; (19) sufficient funds, staff, materials, and time; and (20) skilled leadership [38]. The twenty dimensions can also be summarized as collaborative purpose, member characteristics, communication, process/structure, and environment or resources. At the end of each 1-year term of service, CAB members rate how much they agree or disagree with each of 40 statements on a 5-point Likert-type scale. Scores are averaged across all board members and evaluated according to standards set by the measurement development team. Specifically, an average score above 4.0 demonstrates strong collaboration, averages of 3.0–3.9 are borderline scores, and average scores of 2.9 or below are areas of concern [39]. Following each year, data will be reviewed from this assessment to modify the CAB processes and protocols as needed.

### 2.6. Teacher Assessments

In the *SAGE Teacher Self Evaluation Questionnaire*, an assessment specifically developed for SAGE, teachers rate themselves on how often and how well they complete the PRIME principles when delivering SAGE. Teachers respond to three to five items for each principle (20 items total) using a four-point rating scale of never, rarely, sometimes, or always. Scores are then averaged across all questions within each PRIME principle. At the end of the survey is a section where teachers can write down any topics or areas on which they would such as technical assistance. This questionnaire is filled out during weeks 3 and 10 of SAGE. Week three responses are used to tailor the booster training session to teacher’s specified needs, and week 10 responses help SAGE staff to provide ongoing technical assistance that can enhance sustainability.

The *Teacher Program Sustainability Survey* is based on an existing tool and measures factors associated with program sustainability that are especially associated with the parent-teacher interaction [40]. This 14-item survey is completed once before SAGE starts and once again following delivery of the SAGE curriculum. Questions focus on the types, frequency, and direction of communication between parents and teachers. The survey also specifically asks about parent-teacher communication related to children’s physical activity and nutrition behaviors. Responses from baseline to post-intervention can be compared to evaluate intervention related changes in parent-teacher communication.

### 2.7. Teacher and Director Assessments

*SAGE Self Efficacy Assessment* is an assessment specifically developed for SAGE that is administered to teachers and directors at the teacher training (two to three weeks before start date), the end of the “see one” phase of SAGE (week 5), at the end of the “do one” phase of SAGE (week 10), and following program delivery. This questionnaire includes five statements that staff respond to using a five-point Likert-type scale: (1) strongly disagree, (2) disagree, (3) neither agree or disagree, (4) agree, and (5) strongly agree. Statements ask how confident staff are that they continue to care for and maintain the garden, allow children to water and interact with the garden weekly, provide children with active songs, games, and discussions from the SAGE curriculum weekly, provide children with the mindful eating experience with fresh fruits and vegetables weekly, and engage parents and other community members in SAGE. Averages across all five questions are calculated by staff type (i.e., director vs. teachers) and for each ECEC. Changes or patterns in self-efficacy will be tested to determine whether they mediate changes in other sustainability constructs (e.g., ECEC health environments).

*Interviews.* Exit interviews with ECEC directors, teachers, and other engaged staff (e.g., kitchen personnel, teacher aids) will be conducted after implementation has been completed to investigate factors impacting sustainability along the dimensions of child learning, teacher acquisition of skills needed to deliver the SAGE curriculum, and ECEC achievement of policy guidelines and project milestones. Interview data can be used to inform scaling up SAGE for broader deployment if it is successful in meeting its goals or modifying SAGE if some pieces are unsuccessful. Semi-structured interviews are iterative in nature, taped, transcribed, and analyzed using thematic content analysis. Emergent themes will be identified using a constant comparison approach [41,42,43,44].

### 2.8. Director Assessments

The *Director Program Sustainability Survey* is based on an existing tool [40] and measures factors associated with program sustainability including ECEC administration, policies, and procedures. Specifically, questions focus on the type of ECEC (e.g., for-profit vs. non-profit), the number, types, processes, and rules for meals served at the ECEC; current physical activity and nutrition training offered to staff at the ECEC as well as future training needs; use of best practices for physical activity and nutrition at the ECEC as well as barriers/facilitators to using those practices; and ECEC directors’ communication with parents. Directors complete this survey prior to and following implementation of SAGE. The 38 questions on this survey consist of check-all that apply, multiple choice, fill in the blanks, and Likert-type scale questions. Responses from baseline to post-intervention will be compared to evaluate the impact of the SAP on outcomes.

The *Sustainability Needs Assessment* (SNA) is based on an existing tool [1] and completed by the ECEC director during SAGE week 3 and SAGE week 10. This assessment allows the director to rate the SAGE program on five RE-AIM dimensions: reach, efficacy, adoption, implementation, and maintenance [45]. Each RE-AIM dimension is evaluated using 6–10 questions (37 total), which are scored using a five-point Likert-type scale: (1) strongly disagree, (2) disagree, (3) neither agree or disagree, (4) agree, and (5) strongly agree. The SNA also investigates process factors associated with implementing the new program such as the perceived feasibility of the study design, the center setting, and the community environment (e.g., having the space and resources available to support the program). Responses from baseline to post-intervention will be compared to evaluate the impact of the SAP on SAGE’s perceived RE-AIM characteristics.

### 2.9. Parent Assessments

The *Newsletter Survey* asks parents to evaluate the newsletters and sustainability of SAGE processes. Specifically, the survey includes seven questions that assess SAGE-related parent-teacher and parent-child interactions, the frequency of receiving, value, and preferred aspects of the newsletters, and overall impact of the SAGE program on their child. The survey also contains a space for parents to write testimonials (if applicable) about positive or negative aspects of SAGE.

### 2.10. Environmental Assessments

Three validated *Nutrition and Physical Activity Self-Assessment for Child Care (NAP SACC)* [46] environmental assessments at each ECEC will be used to measure the quality of resources related to physical activity, nutrition, and screen time. Evaluations used a combination of direct observation by research staff, self-report by teachers, and self-report by directors to measure outcomes. About two weeks before the start of the programming (either SAGE Garden or Child Safety), a research team member assesses outdoor play environment quality, ECEC teachers reported on the indoor environment, and the ECEC director reported the quality of physical activity, nutrition, and screen time policies. This 24-item assessment consists of check all that apply and Likert-type scale questions. For a further description of the assessment process, see Szeszulski et al., 2022 [47]. Responses from baseline to post-intervention will be compared to evaluate the impact of the SAP and SAGE on physical activity, nutrition, and screen time environments.

The *Physical Activity Resource Assessment (PARA)* is an established direct observation checklist [48] that is completed by SAGE staff on site at each ECEC two to three weeks before the start of programming. This assessment consists of fill in the blank, multiple choice, yes or no, and Likert-type scale questions. Questions focus on the availability of physical activity features such as sports fields, play equipment, and trails, amenities such as water fountains, lighting, and shade, and incivilities such as broken glass, graffiti, and vandalism. This 45-item assessment takes about 10 min to complete. Responses from baseline to post-intervention will be compared to evaluate the impact of the SAP and SAGE on the physical activity environment.

### 2.11. Other Assessments

Meeting minutes and field notes will be maintained to document concerns about sustainability raised by the CAB, parents, ECEC directors, and teachers. These can be triangulated by level and extent of replicability and institutionalization to qualitatively examine the relationship between sustainability and child outcomes.

Cost to deliver the intervention is estimated in constant dollars using the Prospective Payment System Input Price Index [49]. This index examines (1) costs of identifying and recruiting participants, including ECEC staff, (2) direct intervention labor costs, including teachers’ time, (3) personnel recruitment and training costs, and (4) material and supply costs (e.g., printed material, garden supplies). Care will be taken to accurately separate research-based costs from replication or implementation costs [50]. A cost-to-deliver spreadsheet will be created to abstract information from production of supplies; receipts from supplies; and times for training, delivery, and administration weighted by employment cost. Costs can be factored per participant to determine a cost to deliver the intervention for each participant.

## 3. Discussion

This paper describes the SAP model and process for improving the sustainability and institutionalization of the SAGE program. There are few existing, comprehensive, and theoretically-guided strategies to evaluate sustainability in ECE programming that can be linked to Sustainable Development Goals put forward by the World Health Organization, following the United Nations’ lead, such as improving nutrition and helping to eliminate preventable disease [51]. Gardens and garden-based curricula, if sustained by ECE sites, may help to contribute to a multilevel systems approach to attaining these goals. The SAP is theoretically guided using the EMO [31], an ecologic framework guiding intervention planning and measurement at multiple levels of analysis. The SAP was conceived using sustainability constructs developed during the multi-site and multi-level CORD trial [10]. The result is a comprehensive mixed methods analysis plan relying on both observed and self-reported data, to determine the potential for sustainability of SAGE, a garden-based physical activity and nutrition intervention to prevent obesity in young children linked to best practices and policy for ECECs [34,36].

This study proposes to measure the depth of implementation of various elements of the customizable SAGE curriculum to determine what is sustained, or expanded upon in future iterations of the programming. A strength of providing a customizable curriculum for sustainability is that *replicability* is enhanced [2]. This is particularly important for underserved populations and low-income areas, where resources for delivering evidence-based interventions may be limited. Giving teachers supportive sustainability strategies to access a variety of virtually no cost games, learning opportunities and songs, and low-cost science experiments coupled with the boon of experiential learning provided by a garden is helpful for increasing replicability and scalability. These activities are simple to do, and the addition of a website with instructions, printable materials and video demonstrations can provide opportunities for teachers to pick and choose the activities that they prefer with on demand support available. Future sustainable development of SAGE could include resources to deliver the curriculum in other languages, enhancing access to sustainable learning opportunities for scaling-up SAGE to be offered internationally.

There are few studies about dissemination and implementation research in order to sustain physical activity and nutrition, garden-based programming in ECECs [25,26,52,53,54]. This study is innovative in that it plans for anticipated barriers based on the experience of a CAB as well as a review of the scientific literature [34,35,36], and includes strategies to measure implementation at multiple levels of analysis guided by the EMO [28,29,31]. The inclusion of a knowledgeable scientific team with experience working with, developing, and operationalizing sustainability constructs suitable for measurement in ECEC sites and in connected pathways [1,2,10].

The *continuation of benefits*, from the school to the home, is an important element that should be captured as part of all implementation and sustainability programming [2]. Parent engagement is an important area for improvement in most ECE and school-based intervention programming, including SAGE, which is dependent on communication with parents and families of the child [54]. Although ECECs provide important learning opportunities that young children might not be able to obtain at home, a second important role of these sites is that they are a safe place for parents to leave their young children while they are at work. Measuring parents’ perceptions of outreach materials (i.e., newsletters) is another strength of this study to assess the potential for *continuation of benefits*.

Since the SAGE SAP is comprehensive, one potential limitation is the high intensity of the labor involved to collect information from numerous data sources. This level of intensity can discourage adoption by ECEC personnel and impede *institutionalization* of the programming itself [10]. As previously discussed, even the most carefully crafted plans for evaluation can be derailed by competing priorities (e.g., changing educational policy priorities; teacher strikes) and forces of changes (e.g., pandemics; telecommunication interruptions) [2]. Strong *community capacity*, defined by including a CAB and investing in high ECEC site engagement, provides the ability to seek counsel and resources to support flexibility to pivot to strategies that may be more helpful for implementing programming and completing assessments, such as using online surveys or virtual platforms (e.g., Zoom). In addition, encouraging ECEC personnel to take pictures of site successes (e.g., happy children, thriving garden) and struggles (e.g., garden pests, broken or overused supplies) is another strategy to help enhance sustainability of various elements of the curriculum. These pictures are also a great way to keep study sites connected on social media to strengthen the community support for garden-based programming. Strong communication and good relationships with community partners and on-the-ground implementers is extremely important for improving adoption as well as sustainability [55].

## 4. Conclusions

Sustaining effective, evidenced based programming to increase physical activity and fruit and vegetable consumption in ECE settings can be challenging. Planning for sustainability must include assessment of contextual factors, and the SAP must be included alongside programming in order to assess achievement of sustainability goals and ensure that these goals continue to be met. Teachers face challenges such as lack of knowledge or skills, lack of resources to implement programming, lack of time to cover content, and even lack of interest in active time. These micro-level implementation challenges as well as macro-level policy challenges that affect sustainability of programming underscore the need for multi-level/multi-sector collaboration, which the SAGE SAP includes as a strategy to enhance sustainability. There is a need for sustainable programming that applies best scientific evidence and includes the voices of community partners and practitioners. This is both efficient at overcoming barriers to sustainability as well as enhancing fun for the end users: teachers, students, and families. The SAGE SAP model and process presents a complete solution. It has been designed in collaboration with ECE teachers and parents together with an expert community advisory board, leveraging the best of science and community, and offering a comprehensive model and process for evaluating and ensuring sustainability that capitalizes on these strengths.

## Figures and Tables

**Table 1:** Measures for Evaluating the SAP by Ecologic Model of Obesity (EMO) Level and Sustainability Construct.

EMO Levels	CORD Sustainability Constructs			
	Replicability (repeatability, adaptability, expandability)	Continuation of Benefits (continued infrastructure and resources)	Institutionalization (policies and organizational structures)	Community Capacity (collaboration, community commitment, resource access)
Micro level	Teacher interviews Director Program Sustainability Survey Teacher Self-efficacy Assessment Field notes	Director Program Sustainability Survey Teacher Self-efficacy Assessment Meeting minutes Field notes	NAP SACC PARA Director Program Sustainability Survey Teacher Self-efficacy Assessment Sustainability Needs Assessment	Wilder Collaboration Factors Inventory Sustainability Needs Assessment
Meso level	Teacher interviews SAGE Teacher Self Evaluation Questionnaire	Teacher interviews SAGE Teacher Self Evaluation Questionnaire Parent newsletter survey	N/A	N/A
Exo level	Teacher Program Sustainability Survey Teacher Self-efficacy Assessment	Teacher Program Sustainability Survey Teacher Self-efficacy Assessment	N/A	N/A
Macro level	Meeting minutes	Meeting minutes	NAP SACC	Wilder Collaboration Factors Inventory

Note. NAP SACC, Nutrition and Physical Activity Self-Assessment for Child Care; PARA, Physical Activity Resource Assessment.

**Table 2:** Timing of SAP Assessments.

	Baseline	Week 3	Week 5	Week 10	Post-Intervention
*Community Advisory Board*					
Wilder Collaboration Inventory					X
*Teachers*					
Self-Evaluation		X		X	
Teacher Survey	X				X
SAGE Reflections		X	X	X	
*Directors and Teachers*					
Self-efficacy	X		X	X	X
Interviews				X	
*Directors*					
Director Survey	X			X	X
Sustainability Needs Assessment		X		X	
*Parents*					
Newsletter Survey					X
*Environmental Assessments*					
PARA	X				X
NAP SAC	X				X
*Other Measures*					
Field/Meeting Notes	X	X	X	X	X
Cost to Deliver					X

## Data Availability

No datasets involved in this manuscript.

## Supplementary Materials

The following supporting information can be downloaded at: https://www.mdpi.com/article/10.3390/ijerph19095511/s1. Supplementary File S1: SAGE Garden Build Protocol.
