# Supplementary material for: Sustainability via Active Garden Education: The Sustainability Action Plan Model and Process
Source: Int J Environ Res Public Health. 2022 May 1;19(9):5511. doi: 10.3390/ijerph19095511 (PMC9102810; doi:10.3390/ijerph19095511)
Supplement: Supplementary file 1 [file ijerph-19-05511-s001.zip › ijerph-1639828-supplementary.pdf]

SAGE
Sustainability via Active Garden Education

# Garden Build Protocol

## Supplies Needed

- Tomato Cages
- Stakes (4-5ft. tall)
- Trellis
- Pruners
- Hand Shovels
- Shovel
- Hand cultivators
- Hand Weeder
- Garden Hoses
- Cardboard
- Tape measure
- Watering Can
- Water wand

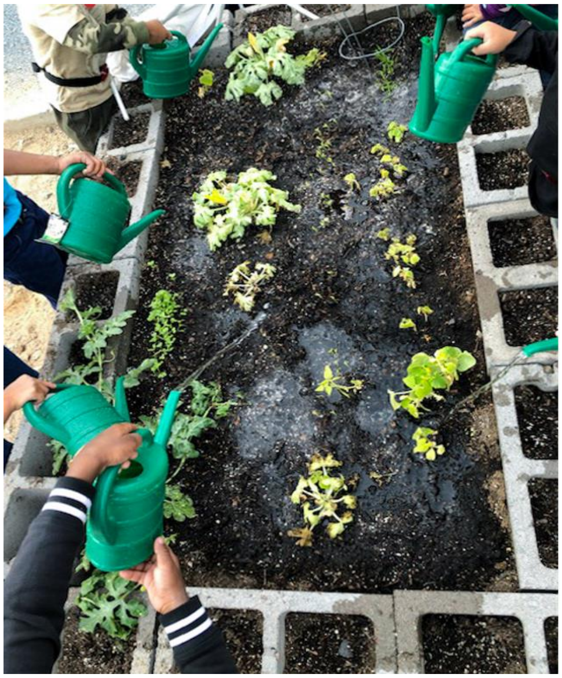

## Instructions

### Step 1

Decide where to build the garden on campus. The following concerns should help you decide where plants will grow best:

- **Sun**. A vegetable garden needs 5-8 hours of full, direct sun every day for plants to be healthy.
- **Water**. Hoses are heavy and often can't be left in the schoolyard. You will want to build your garden as close as possible to a water spigot or hose bib. Or install one near your garden site.
- **Drainage.** Most plants will die if they sit in soggy soil. Make sure that the site you choose isn't the lowest place on campus. Watch where water sits longest after it rains, and you'll know where you don't want to build your garden.
- **Access**. The garden needs to be close enough to classrooms that it can be used regularly. A garden that is out of sight cannot be easily accessed by the students.

SCAN ME

**Edson College of Nursing and Health Innovation**
**Arizona State University**
https://sites.google.com/asu.edu/sageasu/home

**Step 2**

Once you've decided where to build your garden you will want to clear the land and prepare it for your garden build. It is important to start with a clean, flat, weed-free site.

- Using the tape measure and stakes, mark off a six foot by four foot area, using the stakes to mark the four corners.
- Weed the marked off area to remove any growing weeds.
- Level the area using a shovel to prevent drainage problems.
- Cover the leveled area with cardboard in order to prevent weeds from growing up into the garden in the future.

**Step 3**

Now it's time to begin building your garden bed.

- Using the stakes as a guide, begin to place the cinder blocks on the ground in the shape of a rectangle.
    - Length: Four cinder blocks in length.
    - Width: Three cinder blocks in length.
- Create one row of blocks before adding a second row of blocks.
- To the extent possible, overlap the block joints so they do not line up between top and bottom rows.

**Step 4**

The next step is to fill your garden bed with soil.

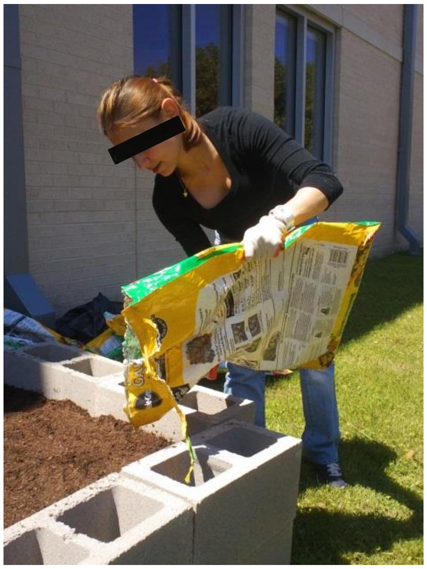

- Open your soil bags and fill the cinder block bed with soil. Use the shovel to evenly distribute the soil.
- Water as you fill the bed with soil to ensure that the soil settles.
- Leave three to five inches at the top of the bed.
- Once the bed is filled, water once again to hydrate the soil.
- Let the bed stand for one week to let the soil settle, watering frequently so that the bed does not dry out.

SCAN ME

**Edson College of Nursing and Health Innovation**
**Arizona State University**
https://sites.google.com/asu.edu/sageasu/home

**Step 5**

Once your garden bed is filled, it is time to start planting.

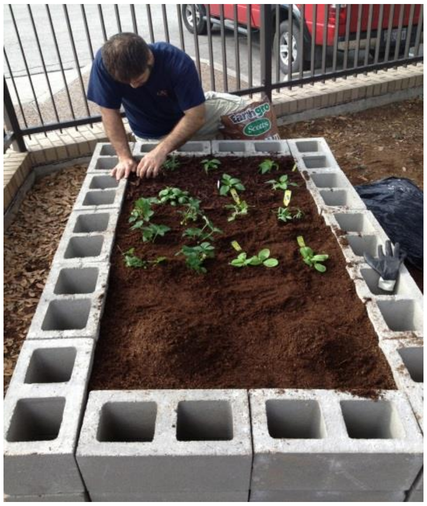

- Purchase desired fruit and vegetable seeds and seedlings.
- Follow the instructions on the package in order to make sure that plants are spaced appropriately and have enough depth for the roots.
- Use a trellis, tomato cages, and stakes for tall plants and plants with vines.

**Step 6**

Now that you've built your garden, it is important to make sure that you maintain your garden so that the plants continue to grow healthy and strong.

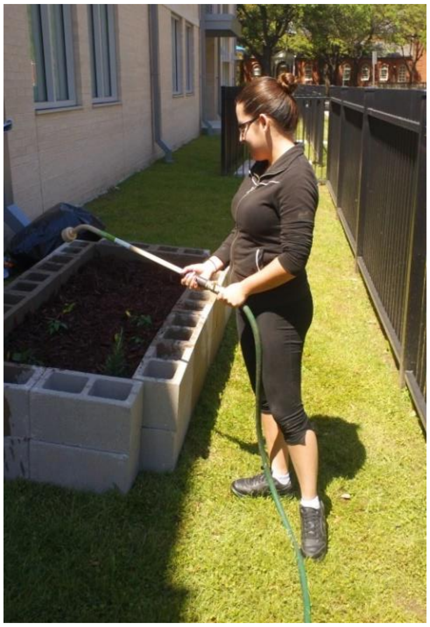

- Cultivate the soil before water when possible. Loose, aerated soil more easily absorbs water.
- Water slowly and gently using the watering can or water wand to water the plants so that the plants are not damaged by the hose's high water pressure.
- Soak the soil deeply, plants will have a hard time developing roots if the only moisture available is on the surface.
- Avoid overwatering the soil. You do not want to flood the plants. Flooding can harm the plants and displace seeds.
- Weeds compete for water, nutrients, space, and light. Remember to weed your garden, removing all parts of the weed, including the roots.
- If you have small shrubs or fines, prune any damaged, diseased, or dead branches at any time.

**Step 7**

Enjoy your garden!

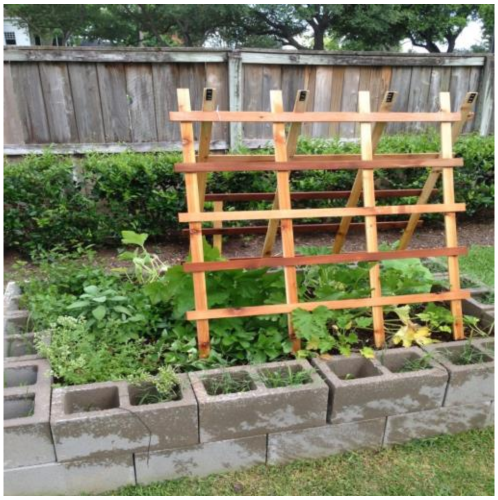

SCAN ME

**Edson College of Nursing and Health Innovation**
**Arizona State University**
https://sites.google.com/asu.edu/sageasu/home
